# Histone deacetylase inhibition activates Nrf2 and protects against osteoarthritis

**DOI:** 10.1186/s13075-015-0774-3

**Published:** 2015-09-26

**Authors:** Dawei Cai, Shasha Yin, Jun Yang, Qing Jiang, Wangsen Cao

**Affiliations:** Jiangsu Key Laboratory of Molecular Medicine, Nanjing University School of Medicine, Nanjing, 210093 People’s Republic of China; Center of Diagnosis and Treatment for Joint Disease, Nanjing Drum Tower Hospital Affiliated with Nanjing University School of Medicine, Nanjing, 210008 People’s Republic of China; Model Animal Research Center of Nanjing University, Nanjing, 210032 People’s Republic of China; National Clinical Research Center of Kidney Diseases, Jinling Hospital, Nanjing University School of Medicine, Nanjing, 210016 People’s Republic of China

## Abstract

**Introduction:**

Osteoarthritis (OA) is a common joint disease that can cause gradual disability among the aging population. Nuclear factor (erythroid-derived 2)-like 2 (Nrf2) is a key transcription factor that regulates the expression of phase II antioxidant enzymes that provide protection against oxidative stress and tissue damage. The use of histone deacetylase inhibitors (HDACi) has emerged as a potential therapeutic strategy for various diseases. They have displayed chondroprotective effects in various animal models of arthritis. Previous studies have established that Nrf2 acetylation enhances Nrf2 functions. Here we explore the role of Nrf2 in the development of OA and the involvement of Nrf2 acetylation in HDACi protection of OA.

**Methods:**

Two OA models—monosodium iodoacetate (MIA) articular injection and destabilization of the medial meniscus (DMM)—were used with wild-type (WT) and Nrf2-knockout (Nrf2-KO) mice to demonstrate the role of Nrf2 in OA progression. A pan-HDACi, trichostatin A (TSA), was administered to examine the effectiveness of HDACi on protection from cartilage damage. The histological sections were scored. The expression of OA-associated matrix metalloproteinases (MMPs) 1, 3, and 13 and proinflammatory cytokines tumor necrosis factor (TNF)-α, interleukin (IL)-1β, and IL-6 were assayed. The effectiveness of HDACi on OA protection was compared between WT and Nrf2-KO mice.

**Results:**

Nrf2-KO mice displayed more severe cartilage damage in both the MIA and DMM models. TSA promoted the induction of Nrf2 downstream proteins in SW1353 chondrosarcoma cells and in mouse joint tissues. TSA also reduced the expression of OA-associated proteins MMP1, MMP3, and MMP13 and proinflammatory cytokines TNF-α, IL-1β, and IL-6. TSA markedly reduced the cartilage damage in both OA models but offered no significant protection in Nrf2-KO mice.

**Conclusions:**

Nrf2 has a major chondroprotective role in progression of OA and is a critical molecule in HDACi-mediated OA protection.

## Introduction

Osteoarthritis (OA) is a common joint disease and the major cause of disability among the aging population. OA is characterized by progressive degradation in articular cartilage, periarticular bone, synovial joint lining, and adjacent supporting connective tissue elements, which eventually results in a loss of joint function [1]. Although many etiological factors contribute to OA disease progression, such as hereditary, metabolism, and mechanical stress [2, 3], the exact mechanism of OA remains unclear. Currently, there are no satisfactory drugs for effective treatment of OA, and total joint replacement has to be considered in severe cases.

Nuclear factor (erythroid-derived 2)-like 2 (Nrf2) is a key transcription factor that regulates the antioxidant defense system. Nrf2 activates its downstream gene expression by controlling the antioxidant response elements (AREs) located in the promoter regions of its target genes, including antioxidative enzyme heme oxygenase 1 (HO-1) and NAD(P)H:quinine oxidoreductase 1 (NQO1) [4]. Nrf2 activity is regulated by various protein modification processes, such as Keap1-mediated ubiquitinated degradation, protein kinase C/mitogen-activated protein kinase (MAPK)-mediated phosphorylation [5, 6], and histone acetyltransferase (HAT)/histone deacetylase (HDAC)-mediated acetylation [7]. Nrf2 acetylation enhances its transcription capacity and downstream target expression and has been shown to confer protection in animal models of inflammation- and oxidative stress-related disease [7, 8].

HDACs can alter the acetylation status of histone and non-histone proteins and can regulate many physiological and pathological processes. Histone deacetylase inhibitors (HDACi) have therapeutic potential in various diseases [9–11]. Inhibition of HDACs causes hyperacetylation of the target proteins and leads to an alteration of gene expression involved in cell differentiation, proliferation, or apoptosis [12]. Mounting evidence demonstrates that HDACi prevent degradation of cartilage in animal models of OA [13–16], suggesting that HDACs have a protective role in OA. However, the molecular mechanisms underlying the action of HDACi in OA have not been fully elucidated.

Because Nrf2 and its downstream proteins are protective in OA-related joint damage and Nrf2 acetylation enhances Nrf2 functions, we hypothesize that Nrf2 acetylation plays an essential role in the protective effects of HDACi in OA. In this study, we explored the role of Nrf2 in the development of OA and the involvement of Nrf2 in the protective effects of HDACi in OA. We used two OA mouse models—monosodium iodoacetate (MIA) articular injection and destabilization of the medial meniscus (DMM)—to test the role of Nrf2 in the progression of OA. We further determined the requirement of Nrf2 in HDACi protection from OA in both MIA and DMM mice. Our results demonstrate that Nrf2 plays a major chondroprotective role in the progression of OA and is a critical mediator in HDACi protection from OA damage.

## Methods

### Reagents

Trichostatin A (TSA), MIA, and mouse recombinant interleukin (IL)-1β were obtained from Sigma-Aldrich (St. Louis, MO, USA). Anti-matrix metalloproteinase (anti-MMP)-13, anti-MMP-3, anti-MMP-1, and anti-histone 3 antibodies were obtained from Santa Cruz Biotechnology (Santa Cruz, CA, USA). Anti-HO-1, anti-NQO1, and anti-Nrf2 antibodies were purchased from Bioworld Technology (Nanjing, China). Antiacetylated Nrf2 (K599) was obtained from ImmunoWay Biotechnology (Newark, DE, USA), and antiacetylated histone H3 was purchased from EMD Millipore (Billerica, MA, USA).

### Animals

Nrf2-KO male mice (used with permission from Dr. Yamamoto Masayuki) and their wild-type (WT) littermates at 8–10 weeks of age were used in the experiments. Mice were fed a regular sterile chow diet and allowed access to water *ad libitum*, and they were housed under controlled conditions (25 ± 2 °C; 12-h light-dark cycle). All experimental protocols were approved by the Institutional Animal Care and Use Committee (IACUC) of Nanjing University Medical School and were carried out in accordance with Nanjing University Medical School IACUC guidelines.

**Fig. 1:**
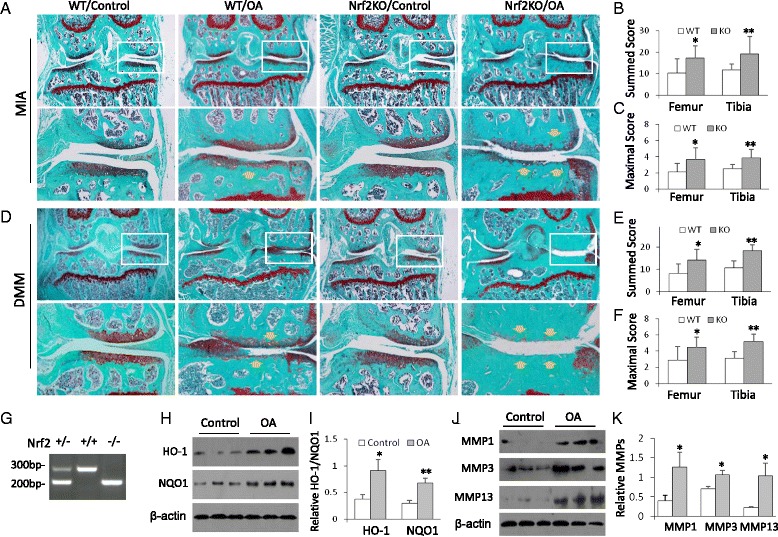
Nrf2-KO mice display more severe articular cartilage damage in osteoarthritis (OA) animal models. **a** Nrf2-KO and wild-type (WT) mice were subject to monosodium iodoacetate (MIA) OA. Mouse joint sections were stained with Safranin O/Fast Green. Representative images are shown (n = 10 mice per group). *Lower panels* show amplifications of *upper panel insets. Yellow arrows* point to the damage-affected cartilage areas. **b** Summed scores of femur and tibia damage in WT and Nrf2-KO mice in the MIA model. *p < 0.05, **p < 0.01. **c** Maximum scores of femur and tibia damage from WT and Nrf2-KO mice of MIA model. *p < 0.05, ** *p* < 0.01. **d** Nrf2-KO and WT mice were subject to DMM surgery. Mouse joint sections were stained with Safranin O/Fast Green and the representative images were shown (n = 10 mice per group). Lower panels were the amplifications of windows from upper panels. The yellow arrows indicate the damage-affected cartilage areas. **e** Summed scores of femur and tibia damage from WT and Nrf2-KO mice of DMM model. *p < 0.05, **p < 0.01. **f** Maximum scores of femur and tibia damage from WT and Nrf2-KO mice of DMM model. *p < 0.05, ** *p* < 0.01. **g** PCR genotyping of WT and Nrf2-KO mice on mouse tail DNA. **h** Western blot analysis of heme oxygenase 1 (HO-1) and NAD(P)H:quinine oxidoreductase 1 (NQO1) from mouse joint tissues of WT and Nrf2-KO mice (three mice in each group). **i** Quantitative analysis of HO1 and NQO1 protein levels. *p < 0.05, **p < 0.01. **j** Western blot analysis of matrix metalloproteinases MMP-1, MMP-3, and MMP-13 from the joint tissues of WT and Nrf2-KO mice (three mice in each group). **k** Quantitative analysis of MMP-1, MMP-3, and MMP-13 protein levels. *p < 0.05. *KO* knockout, *Nrf2* nuclear factor (erythroid-derived 2)-like 2

Genotypes of WT and Nrf2-KO mice were confirmed by polymerase chain reaction (PCR) of genomic DNA isolated from mouse tail (Fig. 1g). PCR amplification was carried out by using one common forward primer, a WT-specific reverse primer, and an Nrf2-KO–specific reverse primer in one PCR. The primer sequences are listed in Table 1.

**Table 1:** RT-PCR and qRT-PCR primers

Genes	RT-PCR primers
MMP-1	Forward: GTCAGGGGAGATCATCGG
MMP-1	Reverse: GCCCAGTACTTATTCCCT
MMP-3	Forward: ATGCCCACTTTGATGATGATGAAC
MMP-3	Reverse: CCACGCCTGAAGGAAGAGATG
MMP-13	Forward: GCTTAGAGGTGACTGGCAA
MMP-13	Reverse: CCGGTGTAGGTGTAGATAGGAA
GAPDH	Forward: CTGCCGTCTAGAAAAACC
GAPDH	Reverse: CCAAATTCGTTGTCATACC
Nrf2GT	Forward: TGGACGGGACTATTGAAGGCTG
Nrf2-WT	Reverse: CGCCTTTTCAGTAGATGGAGG
Nrf2-KO	Reverse: GCGGATTGACCGTAATGGGATAGG
Genes	qRT-PCR primers
IL-1β	Forward: ATGGCAGAAGTACCTAAGCTCGC
IL-1β	Reverse: ACACAAATTGCATGGTGAAGTCAGTT
TNF-α	Forward: ATGAGCACAGAAAGCATGATCCGC
TNF-α	Reverse: CCAAAGTAGACCTGCCCGGACTC
IL-6	Forward: ACACACTGGTTCTGAGGGAC
IL-6	Reverse: TACCACAAGGTTGGCAGGTG
MMP-1	Forward: GCCACAAAGTTGATGCAGTT
MMP-1	Reverse: GCAGTTGAACCAGCTATTAG
MMP-3	Forward: ATGAAAATGAAGGGTCTTCCGG
MMP-3	Reverse: GCAGAAGCTCCATACCAGCA
MMP-13	Forward: ATGCATTCAGCTATCCTGGCCA
MMP-13	Reverse: AAGATTGCATTTCTCGGAGCCTG
NQO1	Forward: TTTAGGGTCGTCTTGGCA
NQO1	Reverse: GTCTTCTCTGAATGGGCCAG
HO-1	Forward: ACATCGACAGCCCCACCAAGTTCAA
HO-1	Reverse: CTGACGAAGTGACGCCATCTGTGAG
β-actin	Forward: TGACGGGGTCACCCACACTGTGCCCATCTA
β-actin	Reverse: CTAGAAGCATTTGCGGTGGACGATGGAGGG

*GAPDH* glyceraldehyde 3-phosphate dehydrogenase, *HO-1* heme oxygenase 1, *IL* interleukin, *MMP* matrix metalloproteinase, *NQO1* NAD(P)H:quinine oxidoreductase 1, *Nrf2* nuclear factor (erythroid-derived 2)-like 2; *Nrf2-KO* Nrf2-knockout, *qRT-PCR* quantitative real-time polymerase chain reaction, *RT-PCR* reverse transcription polymerase chain reaction, *TNF* tumor necrosis factor, *WT* wild type

### Models of osteoarthritis

#### MIA model

The MIA model of OA was adapted from a previous study [17]. Briefly, the mouse right knee joint was flexed at a 90-degree angle, and 6 μl of 5 mg/ml MIA dissolved in sterile saline (0.9 %) was injected into the joint capsule with a 30-gauge needle. Intra-articular injection of saline into the left knee was performed as a control. Three weeks later, the mice were killed for joint and other analysis.

#### DMM model

The experimental mice were anesthetized with peritoneal injection of ketamine (50 mg/kg), and DMM surgery was performed with a microsurgical knife on the right knee by sectioning of the medial meniscotibial ligament (MMTL) that anchored the medial meniscus (MM) to the tibial plateau. The lateral meniscotibial ligament was identified and protected during the surgery. Contralateral knee joints received sham surgery in which the MMTL was exposed without sectioning. Mice were allowed completely free movement following DMM surgery. Six weeks later, the mice were killed and their knee joints were harvested for histological analysis and other examinations.

### Cell culture

SW1353 human chondrosarcoma cells were grown in Dulbecco’s modified Eagle’s medium supplemented with 10 % fetal bovine serum and 100 U/ml penicillin. Cells were maintained at 37 °C in a humidified incubator under a 5 % CO_2_ atmosphere. Cells were treated with different doses of TSA or in combination with IL-1β and then further examined.

### Treatment of TSA

TSA was administrated by subcutaneous injection daily (2.0 mg/kg) for 3 weeks in the MIA model and for 6 weeks in the DMM model. TSA was dissolved in alcohol initially and then diluted with phosphate-buffered saline (PBS) to a final working concentration of 0.1 μg/μl before use [9, 10]. Control mice received the same amount of PBS injection. At the end of the experiment, the mice were killed with CO_2_ inhalation and their knee joints were collected and stored at −80 °C until further analysis.

### Histological assessment

Mouse knee joints were prepared for histological assessment. Briefly, the knee joints were prepared by removing skin and muscle and then immediately fixed in 4 % paraformaldehyde. The knee joints were further decalcified in 20 % formic acid and embedded in paraffin. Frontal serial sections (5 μm thick) across entire joints were obtained, and 10 slides per joint at every 50 μm were selected and stained with Safranin O/Fast Green to assess cartilage destruction.

The Osteoarthritis Research Society International (OARSI) scoring system (grade 0: surface intact, cartilage intact; grade 1: surface intact; grade 2: surface discontinuity; grade 3: vertical fissures; grade 4: erosion; grade 5: denudation; grade 6: deformation) was used to assess the joint cartilage degeneration [18]. The maximum score (the highest score in either quadrant of femur or tibia) and the summed score (the sum of the highest scores in all four quadrants of knee joint: medial femoral condyle, lateral femoral condyle, medial tibial plateau, and lateral tibial plateau) were used to measure the extent of cartilage destruction [18, 19].

### RT-PCR and qRT-PCR

Total RNA was extracted from cultured chondrocytes using TRIzol reagent (Invitrogen, Carlsbad, CA, USA). cDNA was synthesized from total RNA using a reverse transcriptase cDNA synthesis kit (Vazyme Biotech, Piscataway, NJ, USA) according to the manufacturer’s recommendations. The cDNA was amplified by PCR using specific primers and Taq DNA polymerase (Vazyme Biotech). PCR was preceded for 30–35 cycles in a TC-412 thermocycler (Techne/Bibby Scientific, Stone, UK).

Quantitative real-time PCR (qRT-PCR) was performed with cells and joint tissues. Total RNA was extracted from mouse knee cartilage following homogenization in TRIzol reagent. Reverse transcription was performed with l μg of RNA in a total 20-μl reaction using PrimeScript RT Master Mix (Clontech Laboratories, Mountain View, CA, USA) according to the manufacturer’s instructions. qRT-PCR experiments were done with a 7500 real-time PCR system (Applied Biosystems, Waltham, MA, USA) using SYBR Premix Ex Taq reagents (TaKaRa, Shiba, Japan). All data were normalized to β-actin. All assays were done in triplicate. The specific primer sets for qRT-PCR are listed in Table 1.

### Cartilage extraction and Western blot analysis

Cartilage tissue was removed surgically and pulverized in liquid nitrogen shortly thereafter, then stored at −80 °C until analysis. For Western blot analysis, the cartilage tissue was extracted in PBS containing 1 % Triton X-100, 0.1 % SDS, 20 nM sodium orthovanadate, 1 μg/ml aprotinin, 1 mM phenylmethylsulfonyl fluoride, and 5 mM ethylenediaminetetraacetic acid. The homogenates were centrifuged at 12,000 × *g* for 30 minutes at 4 °C, and the protein concentration in the supernatant was measured using a bicinchoninic acid assay kit (Pierce Biotechnology/Thermo Scientific, Rockford, IL, USA), Equal amounts of protein from each animal cartilage were solubilized in lysis buffer containing 10 mM Tris-HCl (pH 7.5), 1 mM ethylene glycol tetraacetic acid, 1 mM MgCl_2_, 1 mM sodium orthovanadate, 1 mM dithiothreitol, 0.1 % mercaptoethanol, 0.5 % Triton X-100, and protease inhibitor cocktail. Proteins were separated by SDS-PAGE and transferred onto a polyvinylidene difluoride membrane. The membranes were blocked with 5 % milk resolved in Tris-buffered saline–Tween 20 buffer, followed by Western blotting with antibodies at predetermined dilutions.

### Luciferase assays

An HO-1 promoter–luciferase reporter plasmid [20] was used to test Nrf2 signaling activity. HEK293 cells were cotransfected with the reporter plasmid and a Renilla luciferase reporter plasmid by using Lipofectamine 2000 reagent (Invitrogen). On the next day, the cells were treated with TSA for 12 h, and a luciferase assay system (Promega, Madison, WI, USA) was used to measure luciferase activity. The relative luciferase activity in each sample was determined by subtracting the background firefly luciferase activity and normalized to Renilla luciferase activity. All reported assays were repeated at least three times independently.

### Statistical analysis

Data were expressed as mean ± standard deviation. Differences between groups were compared by Student’s *t* test or Mann-Whitney *U* test. Analyses were performed using GraphPad Prism 4.0 software (GraphPad Software, La Jolla, CA, USA). A *p* value <  0.05 was considered statistically significant. A *p* value <  0.01 was considered statistically very significant.

## Results

### Nrf2-KO mice display accelerated progression of articular cartilage destruction in MIA and DMM models

To investigate whether Nrf2 mediates HDACi protection against OA, we first tested the role of Nrf2 in the development of OA in the MIA model. WT and Nrf2-KO mice were genotyped by PCR (Fig. 1g). Both WT and Nrf2-KO mice (10 mice in each group) received joint injections of MIA for 3 weeks before being killed. The joint sections were stained with Safranin O/Fast Green. Both WT and Nrf2-KO mice developed arthritis characterized by loss of joint cartilage and exposed subchondral bone with dense sclerosis. Compared with WT mice, Nrf2-KO mice showed much severer cartilage damage (Fig. 1a). To quantify the severity of the joint damage, we analyzed histopathological changes using the OARSI scoring system. WT mice displayed summed arthritic scores of 10.40 ± 6.58 for the femur and 11.75 ± 2.79 for the tibia. The summed scores of Nrf2-KO mice were significantly higher at 17.25 ± 5.76 for the femur and 19.20 ± 8.21 for the tibia (*p <  0.05, **p <  0.01) (Fig. 1b). WT mice had maximum scores of 2.15 ± 1.00 for the femur and 2.50 ± 0.55 for the tibia. The maximum scores of Nrf2-KO mice were also significantly higher (3.65 ± 1.45 for the femur and 3.85 ± 1.05 for the tibia; *p <  0.05, **p <  0.01) (Fig 1c).

To confirm the importance of Nrf2 in the development of OA, we used another model of OA: a DMM model. We performed DMM surgery in the right knee joints of WT and Nrf2-KO mice (10 mice in each group). Six weeks later, the mouse knee joints were subject to histological analysis and the cartilage damage caused by instability was observed in the medial compartment when WT and Nrf2-KO mice were compared (Fig. 1d). WT mice displayed summed arthritic scores of 8.00 ± 4.45 for the femur and 10.65 ± 3.04 for the tibia. In contrast, Nrf2-KO mice showed significantly higher summed scores, with 14.15 ± 4.81 for the femur and 18.35 ± 2.56 for the tibia (*p <  0.05, **p <  0.01) (Fig. 1e). WT mice had maximum scores of 2.85 ± 1.70 for the femur and 3.15 ± 0.74 for the tibia. The maximum scores of Nrf2-KO mice were also significantly higher (femur 4.45 ± 1.25, tibia 5.15 ± 0.98; *p <  0.05, **p <  0.01) (Fig. 1f).

We next examined the expression of two Nrf2 downstream proteins: HO-1 and NQO1. The expression of HO-1 and NQO1 was enhanced in OA cartilage (Fig. 1h, i), indicating that the Nrf2 signaling pathway was activated in OA. In addition, the expression of MMP-1, MMP-3, and MMP-13 was also increased in OA cartilage compared with the control cartilage (Fig. 1j, k). Taken together, these results indicate that Nrf2 is chondroprotective during the development of OA.

### HDAC inhibition activates Nrf2 signaling pathway in chondrocytes in vitro

**Fig. 2:**
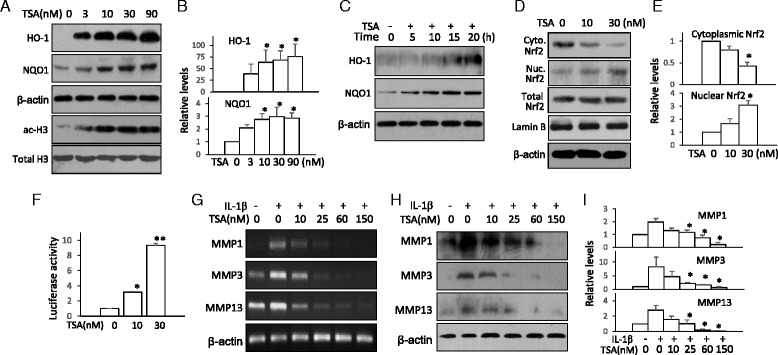
Trichostatin A (TSA) activates nuclear factor (erythroid-derived 2)-like 2 (Nrf2) and suppresses matrix metalloproteinase (MMP) expression in human chondrocytes. **a** SW1353 chondrosarcoma cells were treated with various doses of TSA for 16 h. Heme oxygenase 1 (HO-1), NAD(P)H:quinine oxidoreductase 1 (NQO1), and acetylated histone 3 (ac-H3) were assayed by Western blotting. β-actin and total histone 3 were used as loading controls. **b** Quantitative analysis of HO-1 and NQO1 protein levels. *p < 0.05 compared with control. **c** SW1353 chondrosarcoma cells were treated with TSA (10 nM) for various times. HO-1 and NQO1 were assayed by Western blotting. **d** SW1353 chondrosarcoma cells were treated with TSA (10 nM) for 4 h. The cell cytoplasmic and nuclear fractions were isolated and assayed for Nrf2 by Western blotting. β-actin, total Nrf2, and lamin B from total cell lysates served as controls. **e** Quantitative analysis of cytoplasmic and nuclear Nrf2 from (d). *p < 0.05 compared with no-treatment controls. **f** HEK293 cells were transiently transfected with HO-1 promoter reporter plasmid and a Renilla luciferase plasmid for 24 h. The cells were then treated with 10 nM and 30 nM of TSA for 24 h. The luciferase activity was measured. The reporter luciferase activities were normalized to Renilla luciferase activity. *p < 0.05, **p < 0.01 compared with no-treatment control. **g** SW1353 chondrosarcoma cells were treated with increasing concentrations of TSA for 1 h and then stimulated with interleukin (IL)-1β (5 ng/ml) for 16 h. MMP-1, MMP-3, and MMP-13 were assayed by RT-PCR. **h** MMP-1, MMP-3, and MMP-13 were assayed by Western blotting. **i** Quantitative analysis of MMP-1, MMP-3, and MMP-13 levels from (h). *p < 0.05 compared with IL-1β treatment only. All the Western blotting and RT-PCR results are representative of, and the statistical analyses were performed with results from, at least three independent experiments

To test whether HDACi regulate Nrf2 signaling in chondrocytes, we examined the effect of TSA on the expression of Nrf2 downstream proteins in human chondrosarcoma SW1353 cells. The results showed that TSA induced the expression of the Nrf2 downstream proteins HO-1 and NQO1 in a dose- and time-dependent manner (Fig. 2a–c). TSA also induced marked histone 3 acetylation, as expected (Fig. 2a, lower panel). TSA treatment caused Nrf2 nuclear translocation (Fig. 2d, e) and activated the HO-1 promoter transactivation activity (Fig. 2f). To examine the effect of HDACi on OA-related protein expression, we used IL-1β to induce MMP expression in SW1353 cells. The results showed that TSA treatment suppressed IL-1β–induced MMP-1, MMP-3, and MMP-13 expression at both the mRNA and protein levels (Fig. 2g–i), indicating that HDACi could effectively activate Nrf2 signaling and suppress OA-related MMP expression in chondrocytes.

### TSA alleviates OA-related cartilage damage

**Fig. 3:**
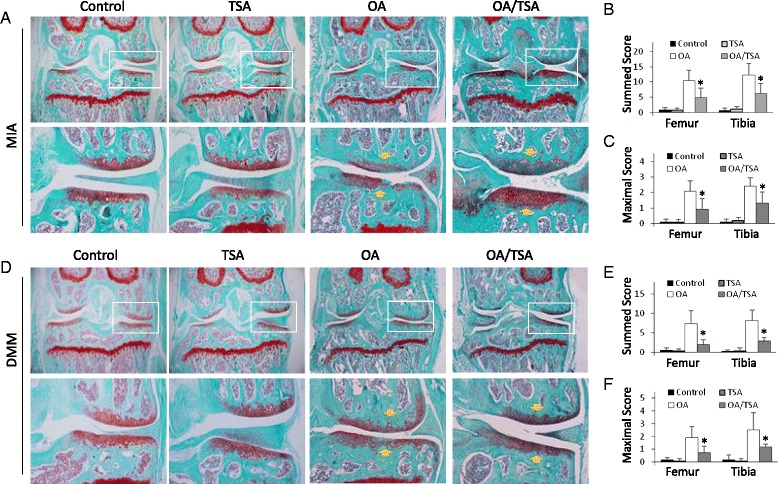
Trichostatin A (TSA) attenuates cartilage damage in monosodium iodoacetate (MIA) osteoarthritis (OA) and destabilization of the medial meniscus (DMM) OA models. **a** Histology of joints in the MIA OA model. Osteoarthritis was induced in mice by MIA articular injection. TSA was delivered via subcutaneous injection (2.0 mg/kg/day). Three weeks later, the joint sections were stained with Safranin O/Fast Green. Representative images of mouse joints are shown (n = 6 mice per group). *Lower panels* show amplifications of *upper panel insets. Yellow arrows* indicate the damage-affected cartilage areas. **b** Summed scores for femur and tibia. *p < 0.05. **c** Maximum scores for femur and tibia. *p < 0.05. **d** Histology of joints in the DMM model. Wild-type and nuclear factor (erythroid-derived 2)-like 2–knockout mice were subjected to DMM. TSA was administrated daily (2.0 mg/kg/day) for 6 weeks. The mouse joints were stained with Safranin O/Fast Green. Representative images are shown (n = 6 mice per group). *Lower panels* show amplifications of *upper panel insets. Yellow arrows* indicate the damage-affected cartilage areas. **e** Summed scores for femur and tibia. *p < 0.05. **f** Maximum scores for femur and tibia. *p < 0.05

To determine the protective property of HDACi on OA, we first tested the effect of TSA in the MIA model. TSA treatment alone did not cause an observable arthritis-like change to mouse knees (Fig. 3a). We injected MIA into mouse knees in the presence or absence of TSA (2 mg/kg/day subcutaneously) for a period of 3 weeks. We then examined the knee joint histology by Safranin O/Fast Green staining. TSA significantly decreased the cartilage damage in the medial tibial plateau and femoral condyle (Fig. 3a, *arrowheads*). To better observe the tide mark and chondrocyte proliferation (cell clusters), we also performed hematoxylin and eosin staining, which clearly showed that the location of the tide mark in the MIA group was much closer to the articular surface, especially in the lateral femoral condyle of both femur and tibia, whereas the TSA-treated group displayed a much improved tide mark location and cell clusters (data not shown). Overall, the MIA group had summed scores of 10.33 ± 3.59 for the femur and 12.25 ± 3.84 for the tibia, whereas the TSA-treated group exhibited significantly lower values of 4.83 ± 3.13 for the femur and 6.17 ± 3.27 for the tibia (*p* <  0.05) (Fig. 3b). When the maximum score analysis was applied, TSA treatment demonstrated similar improvements. The MIA group had maximum scores of 2.08 ± 0.67 for the femur and 2.42 ± 0.53 for the tibia, whereas the TSA-treated group had significantly lower values of 0.92 ± 0.67 for the femur and 1.33 ± 0.69 for the tibia (*p* <  0.05) (Fig. 3c).

We also performed the experiments in the DMM model, TSA (2 mg/kg/day subcutaneously) was delivered for 6 weeks following surgical sectioning of the MMTL. TSA markedly relieved the cartilage damage (Fig. 3d). The DMM group had summed scores of 7.33 ± 3.35 for the femur and 8.17 ± 2.73 for the tibia, whereas the TSA-treated group exhibited significantly lower values of 2.00 ± 1.26 for the femur and 2.92 ± 0.84 for the tibia (*p* <  0.05) (Fig. 3e). The DMM group had maximum scores of 1.92 ± 0.84 for the femur and 2.5 ± 1.35 for the tibia, whereas the TSA-treated group had significantly lower values of 0.75 ± 0.48 for the femur and 1.17 ± 0.24 for the tibia (*p* <  0.05) (Fig. 3f). These results indicate that TSA prevention of cartilage damage was not model-dependent.

### TSA improves OA-associated protein expression

**Fig. 4:**
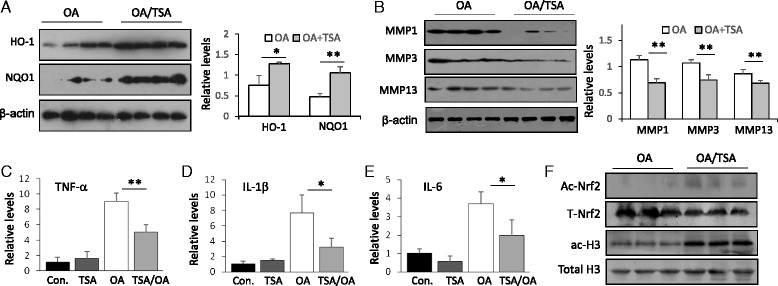
Trichostatin A (TSA) improves the expression of osteoarthritis (OA)-associated proteins and the induction of inflammatory cytokines in monosodium iodoacetate (MIA) OA mice. **a** Western blot analysis of heme oxygenase 1 (HO-1) and NAD(P)H:quinine oxidoreductase 1 (NQO1) from joints of MIA-OA or TSA-treated MIA-OA wild-type (WT) mice. The quantification was done on the right side. *p < 0.05, **p < 0.01 compared with MIA-OA mice. **b** Western blot analysis of matrix metalloproteinases MMP-1, MMP-3, and MMP-13 from joints of MIA-OA and TSA-treated MIA-OA WT mice. The quantification was done on the right side. **p < 0.01 compared with MIA-OA mice. Quantitative real-time polymerase chain reaction analysis of tumor necrosis factor (TNF)-α (**c**), interleukin (IL)-1β (**d**), and IL-6 (**e**) from joint tissues of MIA-OA or TSA-treated MIA-OA WT mice. *p < 0.05, **p < 0.01 compared with MIA-OA mice. **f** Western blot analysis of acetylated nuclear factor (erythroid-derived 2)-like 2 (Ac-Nrf2) and acetylated histone 3 (ac-H3) from MIA-OA or TSA-treated MIA-OA WT mice (three mice in each group). Total Nrf2 and histone 3 served as internal controls. All the Western blot analysis results are representative of, and the statistical analyses were performed with results from, at least six animals

To estimate the involvement of the Nrf2 pathway in TSA protection of OA, we assayed Nrf2 downstream genes *HO-1* and *NQO1*. TSA treatment enhanced the expression of *HO-1* and *NQO1* in MIA mice (Fig. 4a). TSA treatment also suppressed OA-induced MMP-1, MMP-3, and MMP-13 expression (Fig. 4b) and significantly reduced tumor necrosis factor (TNF)-α (Fig. 4c), IL-1β (Fig. 4d), and IL-6 (Fig. 4e) levels. In addition, TSA treatment caused enhanced Nrf2 and histone 3 acetylation in mouse joints (Fig. 4f). These results demonstrate that HDACi-improved cartilage histology is associated with HDACi enhancement of tissue-protective molecules and repression of MMPs and proinflammatory cytokines. TSA protection of OA was correlated with its HDAC inhibition property, as it increased Nrf2 and histone 3 acetylation.

### TSA protection of OA requires Nrf2

**Fig. 5:**
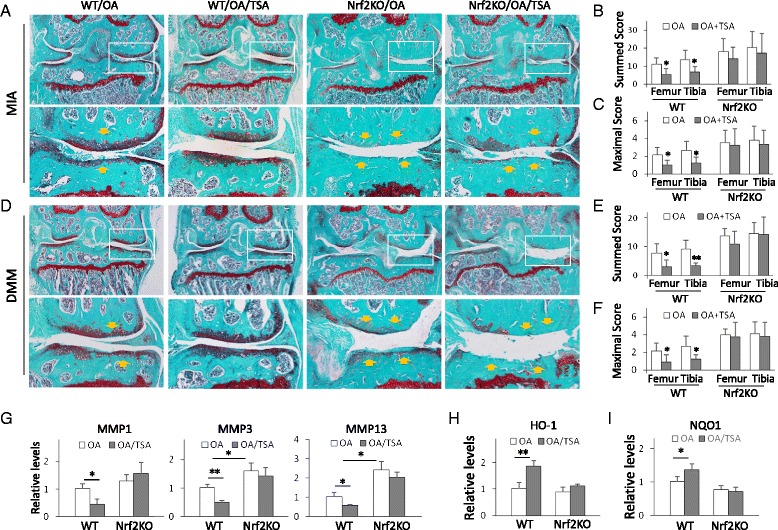
Trichostatin A (TSA) protection of osteoarthritis (OA) requires nuclear factor (erythroid-derived 2)-like 2 (Nrf2). **a** Wild-type (WT) and Nrf2-knockout (Nrf2-KO) mice were subjected to monosodium iodoacetate (MIA)-induced OA or MIA-induced OA with or without TSA treatment (n = 10 in each group). Mouse joint sections were stained with Safranin O/Fast Green. Representative images are shown. *Lower panels* show amplifications of *upper panel insets. Yellow arrows* indicate the damage-affected cartilage areas. **b** Summed scores of femur and tibia damage in WT and Nrf2-KO mice with MIA-induced OA. *p < 0.05. **c** Maximum scores representing femur and tibia damage in WT and Nrf2-KO mice with MIA-induced OA. *p < 0.05. **d** WT and Nrf2-KO mice were subjected to destabilization of the medial meniscus (DMM)-induced OA or to DMM-induced OA with or without TSA treatment (n = 10 in each group). Mouse joint tissue sections were stained with Safranin O/Fast Green. Representative images are shown. *Lower panels* show amplifications of *upper panel insets. Yellow arrows* indicate the damage-affected cartilage areas. **e** Summed scores of femur and tibia damage in WT and Nrf2-KO mice with DMM-induced OA. *p < 0.05, **p < 0.01 compared with OA group. **f** Maximum scores of femur and tibia damage in WT and Nrf2-KO mice with DMM-induced OA. *p < 0.05. **g** Quantitative real-time polymerase chain reaction (qRT-PCR) analysis of matrix metalloproteinases MMP-1, MMP-3, and MMP-13 from joints of WT and Nrf2-KO mice with MIA-induced OA or TSA-treated MIA-induced OA. *p < 0.05, **p < 0.01 compared with MIA-OA mice. qRT-PCR analysis of HO-1 (**h**) and NQO1 (**i**) from joints of WT and Nrf2-KO mice with MIA-OA or TSA-treated OA. *p < 0.05, **p < 0.01 compared with MIA-OA mice

We next decided to determine whether Nrf2 was required for the HDACi protection. We injected MIA into both WT and Nrf2-KO mice and treated mice with TSA as before. In WT mice, TSA significantly reduced cartilage damage, including hypocellularity and loss of proteoglycans; however, TSA failed to reduce the joint damage in Nrf2-KO mice (Fig. 5). Similarly to previous results, WT mice had summed scores of 11.00 ± 3.65 for the femur and 13.50 ± 5.23 for the tibia, whereas TSA treatment led to a significant improvement of 5.33 ± 2.19 for the femur and 6.92 ± 4.24 for the tibia (*p* <  0.05) (Fig. 4b). When the maximum scores were applied, WT mice scored 2.17 ± 0.80 for the femur and 2.67 ± 0.99 for the tibia, whereas TSA showed significantly lower scores of 1.00 ± 0.58 for the femur and 1.25 ± 0.63 for the tibia (*p* <  0.05) (Fig. 4c). However, in Nrf2-KO mice, TSA treatment led to only a marginal reduction of joint cartilage damage (Fig. 5d). Nrf2-KO mice showed summed scores of 18.17 ± 7.20 for the femur and 20.42 ± 8.65 for the tibia, but TSA treatment only slightly reduced the scores to 14.17 ± 6.39 for the femur and 17.33 ± 10.73 for the tibia (Fig. 5b). Nrf2-KO mice had maximum scores of 3.50 ± 1.41 for the femur and 3.83 ± 1.57 for the tibia. TSA treatment was associated with similar scores of 3.25 ± 1.87 for the femur and 3.33 ± 1.57 for the tibia (Fig. 5c), indicating that TSA provided no protection against cartilage damage of OA in mice that lacked Nrf2. These results strongly indicate that TSA protects against cartilage damage in OA through regulation of Nrf2.

We observed similar histological changes in the DMM model (Fig. 5d). Among WT mice, the OA group had summed scores of 7.67 ± 3.35 for the femur and 9.08 ± 3.22 for the tibia, whereas TSA treatment exhibited significantly lower values of 3.00 ± 2.31 and 3.33 ± 0.94, respectively (*p* <  0.05 and *p* <  0.01) (Fig. 5e). The OA group had maximum scores of 2.17 ± 0.90 for the femur and 2.67 ± 1.18 for the tibia, whereas the TSA-treated group had significantly lower values of 0.92 ± 0.79 and 1.25 ± 0.48, respectively (*p* <  0.05) (Fig. 5f). In contrast to TSA alleviation of cartilage destruction in WT mice, TSA treatment neither improved cartilage degradation nor changed the scores significantly in Nrf2-KO mice (Fig. 5e, f, *right panels*).

We also tested the MMP-1, MMP-3, MMP-13, HO-1, and NQO1 mRNA levels in cartilage by qRT-PCR. The results showed that TSA treatment significantly suppressed the induction of MMP-1, MMP-3, and MMP-13 (Fig. 5g) and enhanced the expression of HO-1 (Fig. 5h) and NQO1 (Fig. 5i) in WT mice, but it did not significantly change these levels in Nrf2-KO mice. Consistent with the histopathological changes, Nrf2-KO mice displayed significantly higher levels of MMP-1, MMP-3, and MMP-13 than WT mice, highlighting these MMPs’ destructive role in OA.

## Discussion

HDACi have been used in various animal models of arthritis and are effective in protection against arthritic pathogenesis. Although many contributing factors and/or molecules are proposed to account for the protective mechanisms of HDACi, we found in this study that Nrf2 acts upstream of the signaling and plays an indispensable role in HDACi-mediated protection against OA.

OA is one of the most common aging-related joint diseases, and the molecular mechanisms of OA are not yet fully known [21]. Excessive oxidative stress is associated with OA and triggers the programmed cell death or necrosis of chondrocytes during OA [22]. Proinflammatory cytokines such as TNF-α, IL-1β, IL-6, and MMPs also play major roles in OA pathogenesis [23, 24]. Nrf2 transcription factor is a master regulator of antioxidative stress. Nrf2 signaling regulates the expression of antioxidant-responsive genes and phase II detoxifying enzymes that counteract the oxidative damage in tissue injury [4]. The Nrf2 pathway also crosstalks with inflammatory signaling pathways such as the MAPK and nuclear factor κB pathways and negatively regulates inflammatory responses [25, 26]. Therefore, Nrf2 is expected to provide protection against OA injury. Previous studies demonstrated that Nrf2 was activated in the joints of mice and patients with rheumatoid arthritis and that depletion of Nrf2 accelerated joint inflammation and cartilage destruction in animals with rheumatoid arthritis [27, 28]. Although rheumatoid arthritis and OA share many common features of joint damage, they represent different etiological processes. We employed two OA animal models—namely, DMM and MIA—to verify the critical role of Nrf2 in OA development and progression. The DMM model is one of the most commonly used surgical OA models. In the DMM model, the traumatic injury causes a progressive degeneration of cartilage, subchondral bone sclerosis, and osteophyte formation, which mimic the pathological changes observed in human OA [29, 30]. The MIA model represents the chemical induction of OA by intra-articular injection of MIA. MIA inhibits glyceraldehyde 3-phosphate dehydrogenase activity in chondrocytes and results in cell death following the disruption of the cellular glycolysis process [31]. The cartilage degenerative changes in MIA are comparable to those of human OA, including structural changes such as lack of surface regularities, cartilaginous matrix collapse, and cartilage fibrillation as well as functional changes such as reduction in the compressive stiffness of the articular cartilage [32]. We demonstrated in both DMM and MIA models that Nrf2 downstream proteins HO-1 and NQO1 were upregulated in OA. More importantly, mice lacking Nrf2 developed much more severe joint damage, as judged by histological examination and the summed and maximum score analyses (Fig. 1a–d). These results indicate that, similarly to the mouse model of rheumatoid arthritis, the Nrf2 pathway exerts a major protective function in OA development and pathogenesis.

Recent studies have revealed that Nrf2 is a protein acetylation target. Nrf2 acetylation increases its signaling and enhances its downstream gene expression [7, 8]. We previously showed that HDACi induced Nrf2 acetylation and alleviated the stroke-related brain inflammatory damage [20]. In the present study, we confirmed that the HDACi TSA promoted Nrf2 nuclear translocation, increased expression of Nrf2 target genes *HO-1* and *NQO1*, and activated HO-1 promoter transactivation in chondrocytes (Fig. 2a–d), suggesting that TSA-induced Nrf2 acetylation might contribute to HDACi protection in OA. In the following experiments, we confirmed that TSA enhanced expression of Nrf2 downstream proteins HO-1 and NQO1 in mouse joints and effectively reduced cartilage damage in both MIA- and DMM-treated mice. Cartilage degradation is a hallmark of OA and is caused partially by the excess deposition of cartilage proteoglycan-degrading enzymes such as MMPs [33–35]. Proinflammatory cytokines also promote cartilage injuries [36]. Researchers in previous studies have reported that HDACi repressed MMPs and proinflammatory cytokine expression in joints and reduced the severity of cartilage lesions [13, 14, 37]. We also found that TSA effectively reduced the levels of MMP-1, MMP-3, MMP-13, TNF-α, IL-1β, and IL-6 in mouse joints (Fig. 3). In light of the fact that upregulation of Nrf2 downstream proteins such as HO-1 can reduce the expression of MMPs and inhibit the production of proinflammatory cytokines [38–40], it is reasonable to attribute the inhibition of MMPs and proinflammatory cytokines, at least partially, to the HDACi-induced Nrf2 signaling activation. Also, because HO-1 and the other Nrf2 target proteins are major defenders in tissue repair [41], we speculate that HDACi-induced Nrf2 activation is the major contributor to the improved joint histology. However, it is worth noting that the mice used in our experiments were relatively young (around 8 weeks old) and had immature skeletons. We therefore cannot exclude the possibility that other late developmental events might affect the effectiveness of HDACi and Nrf2 dependency in these two OA models. Future study is needed to examine whether HDACi has similar protective effects in older mice.

To conclusively determine the role of Nrf2 acetylation in HDACi protection against OA, we further compared the effect of TSA on cartilage destruction between WT and Nrf2-KO mice. Our results showed that, although TSA still markedly reduced OA cartilage damage in WT mice, it did not significantly improve the joint injury in Nrf2-KO mice in either the MIA or the DMM model (Fig. 5), indicating that Nrf2 is essential for HDACi-mediated cartilage protection. Previous studies have shown that various types of pan-HDACi and specific HDAC subtype inhibition provide protection against OA injuries [13–16]; however, the acetylation target molecules and the signaling pathways involved are largely unknown. HDACs can either modify non-histone signal molecules, such as Nrf2 in the present study, or target chromatin histones independently to affect gene transcription. HDAC inhibition can also promote signaling molecule association with HAT, bringing HAT to the target gene promoter and subsequently affecting target gene transcription [42]. Our results show that Nrf2-KO mice are not protected by TSA (Fig. 5), suggesting that Nrf2 represents a convergence between Nrf2 acetylation and HDACi actions through which HDAC inhibition provides protection against OA.

## Conclusions

We explored the effectiveness of HDACi in protection against OA in two animal models and identified Nrf2 as the critical molecule mediating HDACi protection. Our study improves understanding of OA pathogenesis and provides novel insights into the development of HDACi-based therapeutic strategies.

## Abbreviations

ac-H3: acetylated histone 3
ARE: antioxidant response element
DMM: destabilization of the medial meniscus
GAPDH: glyceraldehyde 3-phosphate dehydrogenase
HAT: histone acetyltransferase
HDAC: histone deacetylase
HDACi: histone deacetylase inhibitor
HO-1: heme oxygenase 1
IACUC: Institutional Animal Care and Use Committee
IL: interleukin
KO: knockout
MAPK: mitogen-activated protein kinase
MIA: monosodium iodoacetate
MM: medial meniscus
MMP: matrix metalloproteinase
MMTL: medial meniscotibial ligament
NQO1: NAD(P)H:quinine oxidoreductase 1
Nrf2: nuclear factor (erythroid-derived 2)-like 2
Nrf2-KO: Nrf2-knockout
OA: osteoarthritis
OARSI: Osteoarthritis Research Society International
PBS: phosphate-buffered saline
qRT-PCR: quantitative real-time polymerase chain reaction
RT-PCR: reverse transcriptase polymerase chain reaction
TNF-α: tumor necrosis factor alpha
TSA: trichostatin A
WT: wild type
